# The Relationship between Perceived Self-Efficacy and Cervical Cancer Screening among Village Health Volunteers in Suratthani Province, Thailand

**DOI:** 10.31557/APJCP.2021.22.1.179

**Published:** 2021-01

**Authors:** Ornuma Bunkarn, Kiatkamjorn Kusol

**Affiliations:** *School of Nursing, Walailak University, Thasala, Nakhon Si Thammarat, Thailand. *

**Keywords:** Perceived self-efficacy, cervical cancer screening, village health volunteers

## Abstract

**Objective:**

This research aimed to examine the relationship between perceived self-efficacy and cervical cancer screening of village health volunteers.

**Methods:**

The researchers conducted this descriptive research with 279 samples, each recruited using the stratified random samplings. Data were collected in August 2020 using two research instruments included general data and the self-efficacy assessment. The self-efficacy assessment was tested, yielding a reliability score of 0.90. Data were then analyzed using descriptive statistics and point- biserial correlation.

**Results:**

The results revealed that the mean scores of perceived self-efficacy were at a high level in (Mean= 4.35, S.D.= 0.77) and perceived self-efficacy of each has the mean scores of at a high level in strength, generality, and magnitude dimension (Mean= 4.27, S.D.= 0.76; Mean= 4.40, S.D.= 1.01; Mean= 4.35, S.D.= 0.76) respectively. There were significantly positive correlations between the perceived self-efficacy and cervical cancer screening at a very high level (r= 0.81, p <0.001). The relationship between the perceived self-efficacy of each in the strength dimension and magnitude dimension with cervical cancer screening was at a very high level of relationship significantly (r= 0.84, 0.82, p < 0.001). The generality dimension was at a high level of relationship significantly. (r= 0.66, p < 0.001).

**Conclusion:**

This study showed that village health volunteers with high perceived self-efficacy correlated with their confidence screening for cervical cancer. Therefore, village health volunteers should emphasize that women learn and acknowledge the importance of obtaining cervical cancer screening to prevent cervical cancer effectively.

## Introduction

Among all the prevalent health problems in countries worldwide, cancer is the second leading cause of death after coronary heart disease. Statistically, the incidence of new cases is 18.1 million per year and 9.6 million deaths per year. WHO found 60 percent of cancer cases worldwide in Asia (WHO, 2018). In Thailand, cervical cancer has been found the 2nd, following breast cancer; each year, new cases increased by 10,000, about 5 thousand deaths per year. The trend of new cases and deaths constantly climb every year (Ministry of Public Health, Strategy, and Planning Division, 2017). In Surat Thani Province, women aged 15 years and older had recently been diagnosed with cervical cancer at a ratio of 25 cases to 100,000 women (Surat Thani Provincial Health Office, 2018). 

Human papillomavirus (HPV) is what causes cervical cancer, commonly transmitted through sexual contact. Human papillomavirus Type HPV 16 poses the most high-risk as a square cell cervical cancer. Another type referred to as the HPV Type 18 is cervical cancer in the casino type. HPV infection with epithelial cells commences with the HPV entering the basal epithelial cells in a body. As the number of viruses multiplies, p53 protein, and pRb protein (Retinoblastoma protein) was gradually inhibited and destroyed. 

Therefore, HPV-infected cells can cause persistent infection (Maniar and Wei, 2017). The additional factors include premature sex at the age of 16, resulting in a 2.3 times higher risk of developing cervical cancer. Women have had more than ten pregnancy times face seven times as much risk (Kelly et al., 2017). Women not receiving ongoing cervical cancer screening may eventually exhibit signs and symptoms. 

Given the widespread concern, cervical screening is now available free of charge in public health facilities across the country. Besides, a cervical cancer screening campaign for women aged 30-60 launched, emphasizing the pivotal use of condoms and HPV vaccination services for fifth-grade students in primary schools (Ministry of Public Health, Department of Health, 2017). In terms of disease prevention, three widely adopted screening methods for cervical cancer comprise 1) Pap smear, 2) Acetic acid (VIA) test, and 3) HPV test. The standard function by detecting changes in DNA cells was enclosing the cervix (Suksawat et al., 2019). If a cell abnormality is detected, the technician can test the remaining specimens for HPV using HPV DNA testing without collecting new epithelial cells. After that, treatment planning can be continuously carried out (Pradhan et al., 2018).

The National Cancer Institute implements proactive policies and actions to enable people to cooperate to prevent and reduce cancer impacts by raising awareness (National Cancer Institute, 2019). The Ministry of Public Health has also instituted policies and targeted 80 percent of women aged 30-60 years for cervical cancer screening. A referral system was established, allowing physicians to diagnose quickly using the metric criteria. 

In order to reduce cervical cancer incidences in stages 1 and 2, early screening for cervical cancer is required (Ministry of Public Health, Department of Health, 2017). A small number of women aged 30-60 were paid less attention to cervical cancer screening, so the target set did not achieve. Village health volunteers played a significant role in disseminating public health information provided by public health personnel to women and leaders to prevent the disease. Therefore, based on this capacity, the health care team should promote village health volunteers to women to learn from them. Raised awareness and appreciation of cervical cancer screening to prevent cervical cancer and promote health by getting for potential disease development at early stages that have chances of being cured increase as a result (Department of Health Service Support, 2011). It was of paramount importance that the village health volunteers were sufficient knowledge and a positive cervical cancer screening attitude. They were no effect on the screening of women coverage rates for each area. The village health volunteers needed to understand socio-cultural beliefs and enunciated enhancement of women volunteers to augment their knowledge and screen for cervical cancer (Srisuwan et al., 2015). Therefore, the volunteers’ self-efficacy should be promoted confidence in their capacity to overcome the problems and obstacles bolstered.

Previous studies established that marital status, childbirth, perception prevailed as obstacles to screening. Gaining support from neighbors and family is a factor consistently associated with cervical cancer screening (Chansaen et al., 2016). Besides, women also lacked sufficient knowledge, and intimidated staff fears abnormal test results. Other obstacles involve a short time for the examination, and wrong attitude is adopted (Gatumo et al., 2019) It found that women exhibiting profound self-confidence dealt with themselves, which liked how they perceive their abilities to support their decision-making for self-screening for cervical cancer (Sidabutaret al., 2018).Moreover, increased awareness of cervical cancer defined by acknowledgement and intention to seek cervical cancer screening has a strong relationship with self-efficacy development (New-Aaron et al., 2020).

The researchers adopted the self-efficacy theory (Bandura, 1977) in assessing women’s perception of self-efficacy in three principal dimensions: 1. strength dimension, 2. generality dimension, and 3. difficulty dimension to determine the level of expected confidence in village health volunteers’ competency and action. These will give village health volunteers confidence in their cervical cancer screening decision. Therefore, the researchers emphasized investigating the relationship between self-efficacy and cervical cancer screening among the village health volunteers aged 30-60. The researchers believed that if the village health volunteers had high self-efficacy, they would have the ability to choose to get cervical cancer screening. 

## Materials and Methods

This research was descriptive research with a correlation analysis model.

*Population and sample*

The research population was the village health volunteers of women aged 30-60 years in Suratthani, Thailand. 

The sample size was calculated by G*power program 3.1.2 (Hair et al., 2010). The calculation used was convention effect size (Cohen, 1977), with a sample size of 222 people. Therefore, the researchers determined the sample size to increase to prevent 25% of the sample group from decreasing in number during the trial. In the end, a total of samples were 279 samples. 

*Random sample*

Data were analyzed using the SPSS program Version 22. Statistic employed were as follows;

1. Representatives of the village health volunteers aged between 30 and 60 years in Suratthani Province were randomized. 

2. Stratified random sampling applied to one district, namely Phrasaeng District, among all the 19 districts. After that, samples from that particular district, made up of seven sub-districts, were randomly selected which entailed 13 Health Promoting Hospitals.

3. The randomized samples to four Health Promoting Hospitals to represent the sample group in this study.

*Research instruments*

The research instruments consisted of general information and the perceived self-efficacy questionnaire. The self-efficacy characteristics correspond to rating scales. There were 17 questions, divided into seven positive and ten negative questions. The researcher performed calculations based on 30 cases of women aged 30-60 years. Reliability has a confidence value of 0.90, suggesting a reliable assessment. 

*Data collection methods*

Details of the data collection methods were as follows;

1. The research team proposed research projects and research tools to the Human Research Ethics Committee of Walailak University.

2. The research team submitted a form to get permission for data collection through the Dean of School of Nursing, Walailak University, to the Director of the Health Promoting Hospitals. 

3. Before data collection, the researchers, obtained consent from all the research samples. They formally asked for cooperation in the study and clarified details concerning the purpose, research process, benefits and research instruments. 

4. The researchers collected data through questionnaires eliciting general data and the rating scales, which took approximately 30 minutes and validated the completeness of the data obtained before data analysis.

*Statistical Analysis*

Data were analyzed using the SPSS program Version 22. The statistic employed included; 

1. Descriptive statistics to analyze the demographic data, entailing use of frequencies, percentage, mean and standard deviation (SD).

2. Analysis of the relationship between the perceived self-efficacy and cervical cancer screening. The sample analysis performed using Point-biserial correlation coefficient statistics.

*Ethical Approval*

This research was officially approved by the Office of Human Research Ethics Committee of Walailak University as affirmed by the approval letter numbered WU-2-316-63, 21^st^ July 2020. 

## Results

*Sample general data and personal health data*

In terms of the general sample data, 39.4 percent of the participants fell within a 41- 50 age range; the average age was 46.82 years (SD= 7.22), 100 percent were Buddhists. A majority of 79.2 percent was married, 35.8 percent received cervical cancer screening in 2020, 49.8 percent completed primary education, 59.5 percent of occupation was agriculture. A majority of 60.1 percent has served as a village health volunteer for 1 to10 year, 54.5 percent of family economic backgrounds described as having enough to spend, but not to save (Table 1).

A majority did not have a medical history of Vaginitis, Leukorrhea, or Post-intercourse bleeding (73.5%). More than half (63.8 %) had been in labor for 0-2 times. Those are having never taken birth control pills made up a ratio of 35.1. A majority of 63.5 percent of healthcare teams were mainstream supporting persons for cervical cancer screening. 46.0 percent received news about cervical cancer from the health care teams. The reasons for coming to the cervical cancer screening associated with the benefits of examining at 33.7 percent, followed by being intimidated to know about one’s health at 28.7 percent (Table 2).

*Perceived self-efficacy of the sample*

In terms of the mean scores on the perceived self-efficacy of the sample, it found that the sample group had mean scores of self-efficacies in each area in strength dimension, generality dimension, and magnitude dimension in a high level (M= 4.27, SD= .76; M= 4.40, SD= 1.01; M= 4.35, SD= .76). And similarly, the mean score for their overall performance perception is also high (M= 4.35, SD= 0.77) (Table 3).

*Relationship between perceived self-efficacy and cervical cancer screening*

Cervical cancer screening of samples: It found that 264 village health volunteers intend to screen for cervical cancer out of the 279 persons-women with confidence in their abilities. They could offer them some assistance in deciding to be screened for cervical cancer. Turning to an analysis of the relationship between perceived self-efficacy and the samples’ cervical cancer screening, the researchers performed an analysis using Point-biserial correlation coefficient statistics. The analysis indicated a relationship between overall perceived self-efficacy, strength dimension, and magnitude dimension associated with a very high level of cervical cancer screening (r= 0.81, 0.84, 0.82, p< 0.001). The generality dimension is related to a high-level of cervical cancer screening (r = 0.66, p < 0.001) (Table 4).

**Table 1:** General Data of the Study Participants (n= 279)

General data	Number	(%)
Age (Min = 30, Max = 60, Mean = 46.82, S.D. = 7.22)		
30 – 40 year	63	(22.6)
41 – 50 year	110	(39.4)
51 – 60 year	106	(38)
Religion		
Buddha	279	(100)
Marital status		
Married	221	(79.2)
Divorced/Separated	47	(16.8)
Single	11	(4)
Cervical Cancer Screening		
2020	100	(35.8)
2019	84	(30.1)
2018	34	(12.2)
2017	22	(7.9)
2016	10	(3.6)
2015	29	(10.4)
Education level		
Primary education	139	(49.8)
Secondary education	109	(39.1)
Diploma /Bachelor Degree	31	(11.1)
Family economic status		
Enough to spend, not to save	152	(54.5)
Enough to spend, and to save	34	(12.2)
Not enough to pay, no debt	11	(3.9)
Not enough to pay with debt	82	(29.4)

**Table 2:** Personal Health Data of the Study Participants (n= 279)

Personal health data	Number	(%)
History of Vaginitis, Leukorrhea, or post intercourse bleeding		
No	205	(73.5)
Yes	74	(26.5)
Number of labors		
0-2 times	178	(63.8)
3-5 times	92	(33)
5-8 times	7	(2.5)
> 8 times	2	(0.7)
Taking birth control pills		
Never	98	(35.1)
< 5 years	36	(13)
5-10 years	69	(24.7)
> 10 years	76	(27.2)
Mainstream supporting persons for cervical cancer screening		
Health care teams	177	(63.5)
Family person	63	(22.6)
Friend / relative	23	(8.2)
Channels received news about cervical cancer		
Health care teams	250	(46)
Village health volunteers of friend	122	(22.5)
Television	102	(18.8)
Radio	25	(4.6)
Close person	25	(4.6)
Newspaper / Leaflet/ Internet	19	(3.5)
Reasons behind receiving cervical cancer screenings		
The benefits of the examination	94	(33.7)
Know about one's health	80	(28.7
Not want to get cancer in the end stages	64	(22.9)
Trust in the competence of personnel	25	(9)
Easy access to the examination	16	(5.7)

**Table 3:** Mean Scores and Level of Self-Efficacy in Each Dimension and Overall (n = 279)

Perceived self-efficacy	Mean	(SD)	Level
1. Strength Dimension	4.27	(0.76)	High
2. Generality Dimension	4.40	(1.01)	High
3. Magnitude Dimension	4.35	(0.76)	High
Total	4.35	(0.77)	High

**Table 4:** Relationship between Perceived Self-Efficacy and Cervical Cancer Screening (n = 279)

	Cervical cancer screening		
Perceived self-efficacy	R_pbis_	p- value	relationship
1. Strength Dimension	0.84	0.001***	Very high
2. Generality Dimension	0.66	0.001***	High
3. Magnitude Dimension	0.82	0.001***	Very high
Total	0.81	0.001***	Very high

## Discussion

The village health volunteers appraised their perceived self-efficacy from self-efficacy concepts application (Bandura, 1977). This study found that the mean scores were very high overall and in each dimension-likewise, Sidabutar et al., (2018). Women perceived self-efficacy was high because of women’s knowing and preventing cervical cancer self-efficacy, including the economic background that resulted in women becoming aware of cervical cancer screening importance and competency. Furthermore, there was consistency with the village health volunteers’ confidence in strengthening village health volunteers to increase self-efficacy (Nigussie et al., 2019). The village health volunteers should also be aware that cervical cancer cultivates a positive attitude and intention to take care of their health to be free from cervical cancer (Baharum et al., 2020). Therefore, they must perceive their competencies and increase their self-efficacy to empower village health volunteers to strive for self-development.

The relationship between perceived self-efficacy and cervical cancer screening: The sample group screened for cervical cancer (264 out of 279 samples). The results of the relationship between self-efficacy and cervical cancer screening revealed that the overall dimension was very high, statistically significant (r= 0.81, p< 0.001). The relationship between perceived self-efficacy in the three-dimensional and screening for cervical cancer consist of; 1) the strength dimension was playing a role in assessing the confidence in action, 2) the generality dimension was setting the belief in one’s competence, and 3) the magnitude dimension. The study evaluated the village health volunteers in the strength dimension and difficulty dimension with cervical cancer screening. Their associations with very high cervical cancer screening (r= 0.84, 0.82, p< 0.001) were indicated, respectively.

Similarly, for the relationship between the generality dimension and cervical cancer screening, Data suggested an association at a significantly high level (r= 0.66, p< 0.001), elucidating that the village health volunteers with a heightened self-efficacy awareness have the right self-screening decisions for cervical cancer. It was also found that self-taught women were able to overcome barriers to obtain the screening, stemming from their understanding and confidence in their own ability to seek cervical cancer screening (Bucchi et al., 2018). Likewise, Sunisa et al., (2016) pointed out that the village health volunteers’ learning about screening barriers and support by the family were related to the village health volunteers’ cervical cancer screening. Besides, the village health volunteers intended to take care of their health and their confidence in themselves improved; they are knowledgeable about cervical cancer prevention and adopt the right attitude (Gatumo et al., 2019). The women would develop a thinking process that can decide to bolster self-efficacy for cervical cancer screening, which had association with confidence, strength and self-efficacy. They would make it easy for cervical cancer screening (Bunkarn et al., 2020). Therefore, it showed that the village health volunteers’ self-efficacy was highly successful in making a decision to come for cervical cancer screening.

In conclusion, promoting village health volunteers’ development were competencies to have confidence in their cervical cancer screening abilities, including supporting the volunteers to invest their efforts to be aware of the cervical cancer screening importance and decide to come for cervical cancer screening.
